# Short-term physiological effects of pressure rise time modulation during volume-guaranteed neonatal ventilation

**DOI:** 10.1038/s41598-026-44486-5

**Published:** 2026-03-30

**Authors:** Ferid Aliyev, Sule Yigit, Elif Yucel, Umit Ayse Tandircioğlu

**Affiliations:** 1 Division of Neonatology, Department of Pediatrics, Faculty of Medicine, Hacettepe University, Ankara, Turkey; 2 Division of Neonatology, Department of Pediatrics, Faculty of Medicine, Kırıkkale University, Kırıkkale, Turkey

**Keywords:** Mechanical ventilation, Pressure rise time, Cerebral oxygenation, Neonatal physiology, Health care, Medical research, Physiology

## Abstract

Pressure rise time (PRT) is an adjustable ventilator parameter that regulates the rate of inspiratory flow delivery and may influence ventilator mechanics and tissue oxygenation. However, its short-term physiological effects in neonates receiving volume-targeted ventilation remain incompletely characterized. Seventeen hemodynamically stable neonates receiving assist-control volume guarantee (AC-VG) and pressure support volume guarantee (PSV-VG) ventilation were studied using three different PRT settings (0.10, 0.20, and 0.30 s), each applied for 20-minute periods. Ventilator parameters, peripheral oxygen saturation (SpO₂), and cerebral regional oxygen saturation (CrSO₂) measured by near-infrared spectroscopy (NIRS) were continuously monitored. During AC-VG ventilation, modulation of PRT did not result in significant changes in ventilator parameters or oxygenation. During PSV-VG ventilation, peak inspiratory pressure increased with longer PRTs, while mean airway pressure, SpO₂, and CrSO₂ remained unchanged. In this short-term physiological study, modulation of pressure rise time influenced selected ventilator mechanics without producing significant changes in peripheral or cerebral oxygenation under stable clinical conditions. Larger prospective studies incorporating longer observation periods and clinically relevant outcomes are required to further define the role of pressure rise time adjustment in neonatal mechanical ventilation.

Trial registration: This study was retrospectively registered at ClinicalTrials.gov (Identifier NCT07465237).

## Introduction

Despite advances in perinatal care, invasive mechanical ventilation remains an essential intervention in the management of critically ill neonates. While often life-saving, mechanical ventilation is associated with adverse effects on multiple organ systems, including the lungs, cardiovascular system, and brain. The mechanisms underlying ventilator-induced lung injury (VILI) have been largely elucidated through experimental and preclinical studies, identifying both ventilator-related and patient-related contributors to lung injury^1,2^. VILI is now recognized as a multifactorial process arising from the interaction between mechanical forces and the vulnerable neonatal lung.

Among the mechanical forces contributing to ventilator-induced lung injury, volutrauma resulting from excessive tidal volume delivery and alveolar overdistension has been identified as a central mechanism of lung injury. Advances in neonatal ventilator technology have enabled real-time measurement of exhaled tidal volume, facilitating the use of volume-targeted ventilation strategies^3^. Consequently, volume guarantee (VG) has become widely integrated into conventional ventilation modes in neonatal intensive care units, most commonly in synchronized intermittent mandatory ventilation (SIMV), assist-control (AC), and pressure support ventilation (PSV)^4,5,6^.

Although these ventilation modes differ in their triggering and cycling mechanisms, systematic reviews have not demonstrated clear superiority of one mode over another in terms of long-term clinical outcomes^7,8^. This suggests that, beyond the selection of ventilation mode itself, adjustable ventilator parameters within these modes may play an important role in modulating respiratory mechanics and physiological responses in ventilated neonates.

In volume guarantee (VG) ventilation, delivery of the target tidal volume is influenced not only by the selected ventilation mode but also by adjustable parameters that determine the pattern of inspiratory flow. One such parameter is pressure rise time (PRT), which defines the rate at which inspiratory pressure and flow increase at the onset of inspiration. In neonatal ventilators, PRT can be adjusted over a wide range, resulting in either a rapid or more gradual rise to the target pressure and volume.

Despite its routine availability in modern neonatal ventilators, the physiological implications of PRT modulation remain incompletely understood. A shorter PRT results in more rapid inspiratory flow delivery and earlier attainment of peak inspiratory pressure, which may increase mechanical stress on the immature lung, whereas a longer PRT produces a slower pressure rise and altered flow dynamics that may influence ventilator mechanics and gas exchange. However, evidence regarding whether variations in inspiratory flow acceleration, independent of delivered tidal volume, affect ventilator-induced lung injury or systemic physiology in neonates is limited^9^.

In particular, data on the effects of PRT modulation on cerebral oxygenation are scarce. Given the potential for ventilator-driven changes in intrathoracic pressure to influence cerebral perfusion, understanding the short-term physiological impact of PRT adjustment is clinically relevant. Therefore, this study aimed to characterize the short-term physiological effects of varying PRT on ventilator mechanics, peripheral oxygenation, and cerebral oxygenation during volume-guaranteed ventilation in neonates. We hypothesized that modulation of PRT would alter ventilator mechanics, with possible downstream effects on cerebral oxygenation.

## Materials and methods

### Study design and patient population

This prospective, single-center physiological study was conducted in the neonatal intensive care unit of a tertiary university hospital between July 2023 and July 2024. The study protocol was approved by the Hacettepe University Clinical Research Ethics Committee (Approval No: KA-21062, Date: 06/11/2021), and written informed consent was obtained from the parents or legal guardians of all enrolled neonates prior to participation. All methods were carried out in accordance with relevant institutional guidelines and regulations and in compliance with the principles of the Declaration of Helsinki.

Eligible participants were intubated neonates receiving invasive mechanical ventilation in either assist-control volume guarantee (AC-VG) or pressure support volume guarantee (PSV-VG) modes who were considered hemodynamically and respiratorily stable at the time of enrollment. Stability was defined as a fraction of inspired oxygen (FiO₂) requirement below 0.5 and partial pressure of carbon dioxide (PaCO₂) between 40 and 55 mmHg during the preceding 6 h. Hemodynamic stability was defined as the absence of vasoactive medication requirements and the presence of heart rate and mean arterial blood pressure values within the normal reference range for gestational age, without clinically significant fluctuations during the preceding monitoring period. Routine sedative administration is not standard practice for mechanically ventilated neonates in our unit, and no sedative escalation or initiation occurred during the study period. Ventilator settings were stable, with no clinically indicated adjustments made for at least 30 min prior to data collection. All measurements were performed under stable ventilatory and clinical conditions as determined by the treating neonatologist. Infants were eligible if they did not require surfactant therapy or demonstrated normal lung imaging within 6 h following surfactant administration, without evidence of air leak on chest imaging.

Exclusion criteria included hemodynamic or respiratory instability, major congenital cardiac or chromosomal anomalies, known metabolic diseases, or incomplete data acquisition. This relatively stable study population was intentionally selected to allow assessment of ventilator-related physiological effects while minimizing confounding from severe lung disease.

All infants were ventilated using the same neonatal ventilator platform (Dräger VN500, Dräger Medical, Germany). During the study period, patients were continuously monitored by a dedicated neonatal research assistant to ensure physiological stability and to allow prompt intervention in case of clinical deterioration. At the completion of each study phase, data from the mechanical ventilator, near-infrared spectroscopy (NIRS), and pulse oximetry were recorded for subsequent analysis.

As this study was designed as an exploratory physiological investigation to evaluate the short-term, within-subject effects of pressure rise time modulation, no formal a priori sample size or power calculation was performed. The sample size was determined based on feasibility and the availability of clinically stable neonates meeting the inclusion criteria during the study period. Therefore, the relatively small sample size should be considered when interpreting the findings, as the study may have been underpowered to detect subtle but potentially clinically relevant differences in oxygenation parameters, particularly cerebral regional oxygen saturation.

### Interventions and outcome measures

Following establishment of clinically appropriate ventilator settings, all enrolled neonates were monitored under stable conditions for an initial 20-minute baseline period. Ventilator settings were adjusted according to routine clinical practice and included target tidal volume, inspiratory time, respiratory rate, and fraction of FiO₂ in either AC-VG or PSV-VG mode. At baseline, PRT was set at 0.1 s, consistent with standard practice in the unit.

The study employed a prospective, crossover design to evaluate the short-term physiological effects of PRT modulation. The primary outcomes were short-term changes in ventilator-derived parameters and cerebral regional oxygen saturation across different pressure rise time settings. Secondary outcomes included peripheral oxygen saturation and fraction of inspired oxygen requirement. In each ventilation mode, PRT was sequentially adjusted to three predefined levels (0.1, 0.2, and 0.3 s), with each setting maintained for 20 min to allow assessment of short-term steady-state physiological responses. To minimize potential carryover effects, PRT was reset to 0.1 s and maintained for a 30-minute washout period between ventilation modes. Given the rapid physiological equilibration expected following changes in inspiratory flow dynamics during volume-guaranteed ventilation, an additional washout period between sequential PRT adjustments was not considered necessary. The order of ventilation modes (AC-VG or PSV-VG) was randomly assigned for each participant. Randomization was applied only to the sequence of ventilation modes and not to participant group allocation.

During AC-VG ventilation, target tidal volume was held constant and inspiratory time was set at 0.40 s. During PSV-VG ventilation, target tidal volume was similarly maintained, while the maximum inspiratory time was set at 0.43 s.

Throughout each study phase, peripheral oxygen saturation, cerebral regional oxygen saturation measured by NIRS, and ventilator-derived parameters were continuously monitored and recorded. Ventilator pressure–volume and flow–volume waveforms were visually assessed to ensure stability of ventilation. In addition, FiO₂ requirement, mean airway pressure (MAP), and peak inspiratory pressure (PIP) were recorded at each study stage. Blinding was not feasible because ventilator setting adjustments were performed in real time at the bedside during physiological monitoring. Invasive arterial or venous blood gas analyses were not performed, as the study was designed to assess short-term physiological responses under clinically stable conditions.

### Statistical analysis

Statistical analyses were performed using IBM SPSS Statistics version 25.0 (IBM Corp., Armonk, NY, USA). Data distribution was assessed using visual inspection and formal normality testing as appropriate. Descriptive statistics were presented as mean ± standard deviation for normally distributed variables and as median with interquartile range for non-normally distributed variables. The effects of PRT on ventilator parameters and cerebral oxygenation measurements were evaluated using repeated measures analysis of variance (ANOVA). When the assumption of sphericity was violated, the Greenhouse–Geisser correction was applied. Changes in FiO₂ requirement and SpO₂, which did not meet parametric assumptions, were analyzed using the Friedman test. All statistical tests were two-tailed, and a p-value < 0.05 was considered statistically significant.

## Results

A total of 17 neonates (9 females and 8 males) were enrolled. No adverse events were observed during the study period. The median gestational age at birth was 36 weeks (range, 27–39 weeks), and the mean birth weight was 2126 ± 222 g.

All infants were receiving invasive mechanical ventilation at the time of enrollment, and none had confirmed sepsis or congenital anomalies. The mean duration of invasive mechanical ventilation was 17 ± 7 days, and the mean length of hospital stay was 41 ± 8 days. Baseline demographic and clinical characteristics of the study population are summarized in Table 1.

**Table 1:** Neonatal and maternal characteristics of the patients.

Features	Study group, *n* = 17
Gender, female, n (%)	9 (52.9%)
Birth week, w, (range)	36 (27–39)
Birth weight, g, mean ± SD	2126 ± 222
Mode of delivery, CS (%)	16 (94.1%)
SGA, n (%)	3 (17.6%)
Gestational diabetes, n (%)	2 (11.8%)
Premature membrane rupture, n (%)	3 (17.6%)
Preeclampsia, n (%)	1 (5.9%)
Duration of mechanical ventilation, days, mean ± SD	17 ± 7
Length of hospital stay, days, mean ± SD	41 ± 8

CS; cesarean section, SGA; small for gestational age.

Mechanical ventilation in this cohort was required due to physiological respiratory insufficiency rather than primary surfactant deficiency. The clinical indications included postoperative respiratory failure in 9 infants, pneumonia-related respiratory failure in 3 infants, respiratory failure due to impaired central respiratory drive in 4 infants, and sepsis-related respiratory failure in 1 infant. This reflects typical clinical indications for mechanical ventilation in relatively mature or clinically stable neonates in a tertiary neonatal intensive care setting.

Ventilator settings and measured parameters during AC-VG ventilation at different pressure rise times are summarized in Table 2. When inspiratory time (Ti), tidal volume (VT), respiratory rate, and positive end-expiratory pressure (PEEP) were held constant, no statistically significant differences were observed in mean airway pressure, peak inspiratory pressure, or FiO₂ requirement across the three pressure rise time settings (*p* = 0.79, *p* = 0.09, and *p* = 0.16, respectively).

**Table 2:** Ventilator settings and measurements during AC-VG mode with different pressure rise times.

Variable	AC-VG			*P* value
PRT, s	0.1	0.2	0.3	n/a
Ti, s	0.40	0.40	0.40	n/a
VTset (mL/kg)	4	4	4	n/a
RRset (breaths/min)	40	40	40	n/a
PEEP (mbar)	6	6	6	n/a
MAP (mbar), mean ± SD	7.8 ± 1.3	7.7 ± 1.2	7.6 ± 1.1	0.79*
PIP (mbar), mean ± SD	13.5 ± 4.8	13.8 ± 5.3	14.4 ± 5.3	0.09*
FiO_2_ (%), median (IQR)	21 (0)	21 (1)	21 (1)	0.16**
SpO_2_ (%), median (IQR)	99 (6)	98 (6)	98 (5)	0.56**
CrSO_2_ (%), mean ± SD	65.1 ± 2.6	65.6 ± 2.6	65.2 ± 2.8	0.76*

*Repeated measurement analysis of variance, **Friedman test.

PRT; pressure rise time, Ti; inspiration time, VTset; target tidal volume, RRset; set respiratory rate, PEEP; positive end-expiratory pressure, MAP; mean airway pressure, PIP; peak inspiratory pressure, FiO_2_; fractional oxygen concentration, SpO_2_; oxygen saturation, CrSO_2_; brain regional tissue oxygen saturation.

No statistically significant differences in CrSO₂ or SpO₂ were observed across pressure rise time settings during AC-VG ventilation (*p* = 0.76 and *p* = 0.56, respectively).

During PSV-VG ventilation, when maximum inspiratory time, tidal volume, respiratory rate, and positive end-expiratory pressure were held constant, no statistically significant differences were observed in mean airway pressure or FiO₂ requirement across pressure rise time settings (*p* = 0.43 and *p* = 0.93, respectively). Peak inspiratory pressure increased significantly with increasing pressure rise time (*p* < 0.001).

CrSO₂ did not differ significantly across pressure rise time settings during PSV-VG ventilation (*p* = 0.13). Similarly, no statistically significant differences in SpO₂ were observed (*p* = 0.179) (Table 3).

**Table 3:** Ventilator settings and measurements during PSV-VG mode with different pressure rise times.

Variable	PSV-VG			*P* value
PRT, s	0.1	0.2	0.3	n/a
Ti-max/Ti-s	0.43/0.41	0.43/0.42	0.43/0.43	n/a
VTset (mL/kg)	4	4	4	n/a
RRset (breaths/min)	40	40	40	n/a
PEEP (mbar)	6	6	6	n/a
MAP (mbar), mean ± SD	8.0 ± 1.7	7.6 ± 0.9	7.8 ± 1.0	0.43*
PIP (mbar), mean ± SD	13.0 ± 4.6	13.7 ± 5.3	14.7 ± 5.7	< 0.001*
FiO_2_ (%), median (IQR)	21 (2)	21 (1)	21 (3)	0.93**
SpO_2_ (%), median (IQR)	98 (6)	97 (7)	98 (6)	0.179**
CrSO_2_ (%), mean ± SD	65.4 ± 2.8	65.7 ± 2.8	66.4 ± 2.7	0.13*

*Repeated measurement analysis of variance, **Friedman test.

PRT; pressure rise time, Ti-max; maximum inspiration time, Ti-s; spontan inspiration time, VTset; target tidal volume, RRset; set respiratory rate, PEEP; positive end-expiratory pressure, MAP; mean airway pressure, PIP; peak inspiratory pressure, FiO_2_; fractional oxygen concentration, SpO_2_; oxygen saturation, CrSO_2_; brain regional tissue oxygen saturation.

## Discussion

VILI remains a major concern in neonatal respiratory care, despite advances in lung-protective ventilation strategies. While non-invasive ventilation is preferred whenever feasible, invasive mechanical ventilation remains necessary in a substantial proportion of neonates, underscoring the importance of optimizing ventilator settings to minimize ventilator-related injury. In this context, our study examined the short-term physiological effects of PRT modulation during VG ventilation, focusing on ventilator mechanics and oxygenation parameters.

Current understanding of VILI has shifted from barotrauma toward volutrauma as a dominant mechanism of lung injury, with excessive delivered volume playing a central role in alveolar damage^10^. Volutrauma may occur not only at high tidal volumes but also when relatively small additional volumes are delivered to already distended lungs. Other contributing mechanisms, including atelectotrauma related to insufficient positive end-expiratory pressure (PEEP) and oxygen-mediated inflammatory injury, further compound lung damage. Volume-targeted strategies such as VG ventilation aim to mitigate these risks by delivering a preset tidal volume while adapting airway pressures according to lung compliance. Within this framework, inspiratory flow characteristics, including the rate of pressure and flow delivery, represent potentially relevant but incompletely characterized determinants of ventilator-induced physiological effects.

PRT modulates the rate of inspiratory pressure and flow delivery and may therefore influence ventilator mechanics independently of delivered tidal volume. In the present study, modulation of PRT resulted in mode-dependent changes in ventilator parameters. During AC-VG ventilation, variation in PRT did not result in statistically significant changes in mean airway pressure, peak inspiratory pressure, or oxygenation parameters. These findings suggest that, under stable conditions and fixed ventilator settings, short-term adjustment of inspiratory flow acceleration does not substantially alter global airway pressures in this mode.

In contrast, during PSV-VG ventilation, increasing PRT was associated with a significant rise in peak inspiratory pressure, while mean airway pressure and oxygenation parameters remained unchanged. This observation likely reflects compensatory ventilator responses aimed at achieving the preset tidal volume under altered flow conditions. Similar effects of inspiratory rise time on ventilator mechanics have been reported in adult populations with obstructive and restrictive lung disease, where faster pressure rise has been shown to modify inspiratory flow patterns and mechanical workload^11,12,13^. However, data regarding the isolated effects of inspiratory flow acceleration on neonatal ventilator mechanics and physiological responses remain limited.

Our findings are broadly consistent with those reported by Chong et al., who demonstrated that longer pressure rise times were associated with lower mean airway pressure during volume-targeted ventilation^9^. This observation may reflect differences in inspiratory flow delivery, whereby faster attainment of the target volume within a fixed inspiratory time results in higher mean airway pressure.

In the present study, no significant differences in mean airway pressure were observed across pressure rise time settings during PSV-VG ventilation, whereas peak inspiratory pressure increased with longer pressure rise times. This finding suggests that, under volume guarantee conditions, alterations in inspiratory flow acceleration may prompt compensatory increases in pressure to achieve the preset tidal volume, particularly when inspiratory time is patient-determined.

This increase in peak inspiratory pressure likely reflects a combination of ventilator algorithm–driven compensation and patient–ventilator interaction. In PSV-VG mode, inspiratory time and flow profile are influenced by the infant’s spontaneous respiratory effort, while the ventilator dynamically adjusts pressure delivery on a breath-to-breath basis to ensure attainment of the target tidal volume. Prolongation of pressure rise time reduces the rate of early inspiratory pressure and flow delivery, which may delay volume delivery during the initial phase of inspiration. As a result, the ventilator may transiently generate higher peak inspiratory pressures later in the inspiratory cycle to compensate and achieve the preset tidal volume within the available inspiratory time. In addition, variability in spontaneous inspiratory effort and flow demand may further influence pressure delivery, contributing to higher observed peak pressures. These findings are consistent with the adaptive pressure regulation inherent to volume-guarantee ventilation and underscore the interaction between ventilator control algorithms and patient respiratory mechanics in determining pressure profiles.

Differences between our results and those reported by Chong et al. may, at least in part, be related to methodological factors, including variation in the applied pressure rise times and differences in monitoring duration. Additional contributing factors include differences in ventilator platforms, patient maturity, and underlying lung mechanics between study populations. Variations in ventilator flow delivery algorithms and trigger sensitivity may further influence pressure dynamics during PSV-VG ventilation. Moreover, the relatively more mature population in the present study likely exhibited more stable lung compliance and respiratory drive, potentially attenuating the physiological impact of pressure rise time modulation.

The relatively mature gestational age of the study population represents an important consideration when interpreting these findings. Infants born at a median gestational age of 36 weeks are inherently at lower risk for ventilator-induced lung injury compared with extremely preterm neonates. However, this characteristic also constitutes a methodological strength, as it reduces confounding from severe surfactant deficiency and unstable lung mechanics. By studying a more physiologically stable population, the present work was able to isolate the short-term effects of pressure rise time modulation on ventilator mechanics and oxygenation, independent of advanced lung disease. Accordingly, these findings may not be directly generalizable to more immature preterm infants, in whom different physiological responses to inspiratory flow modulation may occur.

Within this context, the absence of significant changes in FiO₂ requirement or peripheral oxygen saturation across pressure rise time settings in both ventilation modes suggests that short-term modulation of inspiratory flow dynamics does not substantially influence oxygenation under stable clinical conditions. It is also possible that the 20-minute observation periods were insufficient to capture more subtle metabolic or regional oxygenation effects. In addition, the volume-guarantee algorithm dynamically adjusts inspiratory pressure to maintain the preset tidal volume, which may have buffered the physiological impact of different pressure rise time settings and rendered PRT a secondary determinant of oxygenation in this relatively stable population.

No clinical deterioration or adverse events were observed across ventilation modes or pressure rise time settings during the study period. To date, limited data are available regarding the physiological and clinical effects of pressure rise time modulation in neonates, and, to our knowledge, only one prior study has examined clinical outcomes associated with different pressure rise time settings^9^. Furthermore, the effects of pressure rise time on cerebral tissue oxygenation have not been previously reported.

In the present study, cerebral regional oxygen saturation did not differ significantly across pressure rise time settings during PSV-VG ventilation. Although a modest, non-significant increase in cerebral oxygenation was observed with longer pressure rise times, this finding should be interpreted with caution, given the exploratory nature of the study and the absence of comparable data in the existing literature. The physiological mechanisms underlying potential effects of inspiratory flow dynamics on cerebral perfusion remain incompletely understood and warrant further investigation.

An additional limitation is the absence of arterial or transcutaneous carbon dioxide measurements during the study periods. Carbon dioxide is a major physiological determinant of cerebral blood flow, and even small changes in PaCO₂ may influence cerebral perfusion independent of oxygenation parameters. Therefore, subtle ventilation-related changes in PaCO₂ may have occurred without being detected by peripheral oxygen saturation or NIRS monitoring. Consequently, although no significant differences in cerebral regional oxygen saturation were observed, we cannot fully exclude the possibility that pressure rise time modulation may have influenced cerebral perfusion through unmeasured changes in carbon dioxide levels.

Similarly, no significant differences in peripheral oxygen saturation were observed across pressure rise time settings in either ventilation mode. Taken together, these findings indicate that short-term modulation of pressure rise time primarily influences ventilator mechanics, while having limited measurable impact on oxygenation parameters under stable clinical conditions.

Although concerns have been raised regarding the potential for prolonged pressure rise times to impair ventilation efficiency, the present findings suggest that extending pressure rise time up to 0.3 s was not associated with adverse short-term effects on cerebral or peripheral oxygenation in this cohort. However, the implications of pressure rise time modulation for lung injury and long-term outcomes, particularly in more immature preterm populations, remain to be determined.

### Limitations

This study has several limitations. First, the sample size was relatively small, reflecting the exploratory and physiological nature of the study. This limited sample size reduces statistical power and may have prevented the detection of subtle but potentially clinically relevant physiological or oxygenation changes. Therefore, the findings should be interpreted with caution and considered hypothesis-generating rather than definitive.

Second, the study was designed to assess short-term physiological responses under stable clinical conditions. Accordingly, invasive blood gas analyses as well as transcutaneous carbon dioxide and oxygen monitoring were not performed, and long-term clinical outcomes such as duration of mechanical ventilation, time to extubation, bronchopulmonary dysplasia, and other neonatal morbidities were not evaluated.

Third, the relatively mature gestational age of the study population limits the generalizability of the findings to more immature preterm infants, who may exhibit different physiological responses to inspiratory flow modulation. Finally, this was a single-center study conducted using a single ventilator platform, which may further limit the external validity of the results.

## Conclusion

In conclusion, pressure rise time represents an adjustable ventilator parameter with potential physiological relevance in neonatal mechanical ventilation. In this short-term physiological study, modulation of pressure rise time influenced selected ventilator mechanics without resulting in significant changes in peripheral or cerebral oxygenation under stable clinical conditions.

Although concerns have been raised regarding the potential impact of prolonged pressure rise times on ventilation efficiency, the present findings do not demonstrate adverse short-term physiological effects associated with extending pressure rise time within the range studied. However, no specific pressure rise time value can be recommended based on the current data. Larger, prospective studies incorporating longer observation periods and clinically meaningful outcomes, particularly in more immature preterm populations, are required to clarify the role of pressure rise time modulation in neonatal respiratory care.

## Data Availability

The datasets generated and/or analyzed during the current study are available from the corresponding author on reasonable request.
